# NEWS – REGIONS

**DOI:** 10.7189/jogh.08.010201

**Published:** 2018-06

## Africa

➢ Patients undergoing surgery in Africa are more than twice as likely to die post-operatively, despite generally being younger, healthier and undergoing more minor surgery, than the global average. This finding arose from a study that collected data on 11 422 adult patients at 247 hospitals across 25 countries, to assess patient outcomes following surgical procedures that required an overnight stay. The number of operations across Africa is very low, and fewer than 43% of surgeries are elective. Just over 18% of all patients developed complications, ranging from stroke to pneumonia, and almost 10% of these patients died – many of these deaths are likely to be preventable. This highlights another problem in Africa's health care – many people who need surgery might not have access to it, due to a shortage of health care workers, hospital beds, and poor systems for patient follow-up post-surgery. Across the continent, there are only 0.7 specialist surgeons, obstetricians and anaethetists per 100 000 population, whilst the recommended figure to reduce the risk of death following surgery is 20-40 per 100 000 population. (The Guardian, 3 January 2018)

➢ Six countries were honoured by the African Leaders’ Malaria Alliance (within the framework of the 30th African Union summit in Addis Ababa) for their remarkable achievements against malaria. This year’s award focused on the impact of reducing malaria incidence and progress towards meeting the World Health Organization (WHO)’s Malaria Global Technical Strategy (GTS). Madagascar, Senegal, Gambia and Zimbabwe’s achievements in reducing malaria by more than 20% between 2015 and 2016 was recognised, and Algeria and the Comoros were recognised for their progress towards implementing GTS for 2020 and reduce malaria incidence by at least 40%. Malaria costs Africa an estimated US$ 12 billion each year in lost productivity, investment and health care costs, and the African Union has committed to eliminating malaria by 2030. According to the WHO, progress against malaria in Africa has been uneven, and in 2016, 15 countries had 80% of global malarial cases – and 14 of these countries were in Africa. (Xinhau, 29 January 2018)

➢ Senegal has cut the numbers of new HIV infections by nearly 75% since 2010, leaving it with one of the lowest prevalence rates in Africa, and bucking the trend of West Africa’s stubbornly-high rate of new infections. Across sub-Saharan Africa, 4.3% of people are HIV-positive, but despite Senegal’s high rate of poverty, only 0.4% of people are HIV-positive. It was the first country in sub-Saharan Africa to start a government-backed ART programme in 1998, which both treats people and lessens their chances of transmitting HIV. In 2003, Senegal’s ART programme became free – several years ahead of the WHO’s recommendation. It has also ensured that drug users and sex workers receive treatment – sex work is legal provided that sex workers have three-monthly health checks, and HIV-positive sex workers can continue working if they receive ART, thus becoming less likely to transmit HIV. This has led to a fall in the prevalence of HIV among sex workers from 28% in 2002 to 7% in 2016. Other measures include free, clean syringes for drug-users, and the involvement of other groups, such as mosques and NGOs, in tackling HIV. Despite these impressive results, 50% of HIV-positive adults and 25% of HIV-positive children remain untreated – and as homosexuality is illegal, gay men are deterred from seeking advice, testing and treatment. (Economist, 1 March 2018)

➢ According to a study from UC San Francisco, Tulane University, the University of Sciences, Techniques and Technologies of Bamako and Mali’s Ministry of Public Health, community health workers (CHWs) who visit door-by-door to identify sick children and offering them free care can cause sharp falls in childhood mortality. The intervention, known as Muso, sent health workers to people’s homes to ask about children’s well-being, provided care at the doorstep and triaged the sickest children to health care facilities. The CHWs provided counselling, malaria diagnosis for people of all ages, plus pneumonia, diarrheal disease and malnutrition treatment for children aged under 5 years. This approach, known as Proactive Community Case Management, costs between US $6-$12 per person/y. Over the 7-year course of the intervention, child mortality in the study area fell to 7 deaths per 1000 live births, compared to Mali’s overall rate of 114 deaths per 1000 births – one of the highest in the world. It focuses on reaching every child as soon as possible – the leading causes of child death are curable, but are “exquisitely time-sensitive”. The researchers are now working on a randomised large-scale scale trial of this approach – this study was not randomised so definitive conclusions cannot be drawn, although it does show that if the fall in Bamko’s child mortality is replicated, then the UN’s goal of reducing child mortality to 25 per 1000 births could be achieved. According to Dr Ari Johnson, one of the study authors and researchers, “this shows us how the end to the childhood mortality crisis is achievable, and how universal health coverage could be achievable, even in some of the most challenging settings. It resets the goal posts of what we think of as possible.” (USCF, 12 March 2018)

➢ Following the Ebola outbreak in 2014, Nigeria reformed its public health agency (the Nigeria Centre for Disease Control – NCDC), transforming its response to infectious diseases – including the current outbreak of Lassa-virus. Since 1 January, 365 people have fallen ill and 81 people have died from Lassa fever, making it the largest recorded outbreak in Nigeria. The NCDC, Nigeria’s first line of defence against disease outbreaks, has grown from 30 doctors in 2011 to more than 130 epidemiologists, microbiologists and other specialists; and is deploying sophisticated data management tools and building diagnostic laboratories to monitor the current outbreak and prepare for the future. The NCDC is co-ordinating its Lassa fever response at an emergency-operations centre modelled on centres deployed during polio outbreaks and the Ebola epidemics. “The Nigeria CDC has become stronger and faster. They came quickly with protective gear, and have sent epidemiologists to detect the source of the outbreak, and to locate the contacts of patients who may have the disease,” said Dr Kingsley Ukwaja, of the Federal Teaching Hospital, Abakaliki in Ebonyi State. However, the NCDC still faces some significant challenges, as many Nigerian states lack facilities to diagnose diseases quickly, making it harder to fight outbreaks from the start. (Nature, 15 March 2018)

## Asia

➢ In Afghanistan, health professionals report significant resistance from village elders and mullahs, particularly in remote areas, towards immunisations – and this resistance could harm children and hinder efforts to eradicate polio. Vaccination resistance is based on the idea that vaccinations contain viruses designed by Western governments to deliberately harm people in the Muslim world. Following extensive global immunisation programmes, polio survives in just three countries – Nigeria, Afghanistan and Pakistan. In Afghanistan, UNICEF vaccinates 5.5 million children aged under 5 years each year, included 1.7 million children from the country’s southern provinces. (*Institute for War and Peace Reporting*, 13 January 2018)

➢ In Bangladesh, high levels of lead have been found in several powdered milk brands – lead is a toxin which is especially dangerous for children, as it can cause immune system complications and delay physical and mental development. The lead was found in 5 out of 15 powdered milk brands, in tests carried out in government laboratories. Dr Md Abdul Aziz, professor at Dhaka Shishu Hospital and a specialist in paediatric surgery, called for immediate government action to avert a public health crisis, noting that lead consumption can cause death, as well as increasing risks of diabetes, cardiovascular disease, obesity, and childhood cancer. Bangladesh has previously acted to warn people about buying certain overseas milk powders, after these were found to be contaminated with melamine in 2008. Following the exposure of high lead levels, the Bangladesh Food Safety Authority ensured that all imported milk powders were tested at designated laboratories before sale. (*Dhaka Tribune*, 9 February 2018)

➢ In rural Bangladesh, more children die from drowning during playtime than any other way, and nationwide deaths from drowning are 10 times higher than the USA – and 15 times higher in rural areas. Johns Hopkins University is working on prevention and surveillance mechanisms to avert these deaths, part of a drowning project funded by Bloomberg Philanthropies. Safety measures include playpens and crèches to keep children from water. In Matlab, drowning deaths have reduced significantly – Matlab has not recorded a single death from drowning amongst children in crèches, and parents suffer less anxiety over their children’s safety. Approximately 50 000 children are now enrolled in more than 2500 crèches across rural Bangladesh. (*Global Health NOW*, 11 April 2018)

➢ Singapore’s government is investing heavily in research programmes in synthetic biology, and is encouraging scientists to make synthetic microorganisms and redesign natural ones, that can be used to produce food, electronics, medicine and energy. In January 2018, it launched a synthetic biology research and development programme, which will receive US$ 19 million over 5 years, and 4 projects have already received funding. Singapore’s synthetic-biology strategy prioritises the development of synthetic cannabinoids, producing rare fatty acids and developing new strains of microorganisms that can be used to create new industrial products, and aims to compete in areas where it already has an edge. Singapore’s interest in synthetic biology has been growing since 2012, when a government task force concluded that the field had commercial potential, and could take advantage of the nation’s existing strengths in biomedicine. The 4 projects that have been funded include 2 that will focus on the sustainable production of cannabis plants for medicine; another project aims to create odd-chain fatty acids, which can be used to make triglycerides found in nutritional supplements, fragrances and food. Singapore is also developing synthetic yeast and bacterial strains for industrial use in producing commercial compounds. Singapore’s biggest advantage over the US in this field is its educated workforce and national strategy, as the US lacks a co-ordinated research programme. (Nature, 25 April 2018)

➢ The family of a deceased 27-year old man, who died in August 2017, has alleged that his death is a result of Japan’s new discretionary work system. The unnamed man worked for 36 consecutive hours from 1.00pm on 4 July to 1.00am on 6 July. He was found dead at home the following month, and a labour bureau ruled that his death was induced by his working conditions, which averaged 87 hours of overtime each month for the two months leading up to his death, exceeding the 80-hour threshold used by authorities to measure death from overwork. Prior to this 2-month period, his monthly overtime was as high as 184 hours. There are concerns that the discretionary work system can lead to longer working hours, and Japan’s Diet is discussing a labour reform bill aimed at addressing Japan’s overwork problem. (*Japan Times*, 17 May 2018)

## Australia and Western Pacific

➢ Research from the International Papillomavirus Society revealed a sharp fall in the rate of Human Papillomavirus (HPV) in women aged under 24 years in Australia, falling from 24% to 1% in 10 years. This is attributed to the roll-out of Australia’s national immunisation programme in 2007, and the new DNA screening test. HPV can lead to cervical cancer, and although about 80% of women are infected with HPV, most do not develop cervical cancer as a result. An estimated 930 women will develop cervical cancer in Australia in 2018, and 258 will die, and globally one women dies from cervical cancer every two minutes. Moreover, the Pacific-Oceania and Asian regions have approximately 50% of all global cases of cervical cancer, and the effectiveness of Australia’s vaccination and screening programmes shows it can be beaten. An improved version of the vaccine used will be made available to all 12 and 13 year-olds olds in 2018, and a new screening programme will be launched in December to improve detection of women at risk of developing cervical cancer. (ABC, 4 March 2018)

➢ Greg Hunt, Federal Health Minister, has ordered an investigation into BUPA, a private health insurer in Australia, over its decision to overhaul its medical gap scheme, which doctors have described as “one big leap towards US-style managed care.” BUPA had told doctors that insured patients will only be eligible for gap cover if they are treated at a BUPA-contracted hospital or day-stay facility, with effect from August 2018. BUPA has rejected comparisons with managed care, saying that doctors will continue to decide what treatment is required, and where it will be provided. Psychiatric, rehabilitation and palliative care service will remain unaffected, as the law requires that some cover is required for these services. Under Australia’s Medical Gap Scheme, hospital doctors – including public hospitals and day-stay facilities without an agreement with BUPA will only be paid at the minimum rate the insurers are required to pay, with patients paying the difference. Dr Michael Gannon, president of the Australian Medical Association, warned that patients must ensure that they are not hit with high out-of-pocket costs, and that they may not be able to see their doctor of choice because of inadequate coverage. (*Sydney Morning Herald*, 6 March 2018)

➢ Fiji’s Ministry of Health and Medical Services has declared an outbreak of the life-threatening meningococcal disease. Recently, Fiji has seen an increase in cases – prior to 2016, there was 1-10 cases each year, increasing in 29 cases in 2017; with 18 cases as at 21 February 2018. Despite these increases, meningococcal disease is rare in Fiji, but without appropriate treatment, up to 50% of people will die, and survivors can suffer from permanent disabilities. In 2017, 14.4% of all people infected with meningococcal disease in Fiji died, compared to the death rate of 0.4-0.6% for dengue fever. (Fiji Sun, 20 March 2018)

➢ New Zealand announced plans to boost aid expenditure in the South Pacific, as China’s influence grows in the region. New Zealand plans to spend an additional US$ 500 million over the next few years in international aid (a 30% increase), with much of it directed at the Pacific, and Foreign Minister Winston Peters said that the so-called “Pacific reset” would make New Zealand safer and more prosperous, noting that the South Pacific was becoming an increasingly contested strategic space. It is not yet specified where the increased aid spending will be targeted, but Mr Peters highlighted that climate change is a priority, saying that it posed an existential threat to some island states. He also announced a plan to increase spending on global diplomatic efforts, including re-opening an embassy in Sweden and adding 50 foreign policy staffers around the world. (*Japan Times*, 8 May 2018)

➢ According to the Jakarta Education Authority, the Jakarta administration has ruled out compulsory vaccination for admission to kindergarten and elementary schools. Its deputy head, Bowo Irianto, said that the decision was taken to ensure that access to school is as open and non-discriminatory as possible. A Jakarta citizen, Widhi Maulana, has criticised the decision, and is still proceeding with immunisation for his two-year old child. “The previous regulation was good to ensure that children are vaccinated, but the administration has changed,” he said in a WhatsApp comment. Bowo Irianto points out that immunisations can be carried out after children have started school, and that the city’s health facilities will carry this out. (*Jakarta Post*, 16 May 2018)

## China

➢ Previously, China’s pharmaceutical industry concentrated on replicating Western drugs, and getting new drugs approved was arduous and time-consuming (indeed, the last time that China produced a new drug for the global pharmaceutical industry was in the 1970s, when Tu Youyou developed artemisinin as a malaria treatment). This led to companies investing in safe revenue streams, as R&D investment was risky. However, China is now playing a bigger role in the global pharmaceutical market, as its government is prioritising pharmaceutical innovation, partly to cope with growing burden of cancer and diabetes in China. China plans to speed up drug approvals, and reverse its scientific brain-drain by luring scientists home. Land, grants, tax breaks and investments in research are also promised. There are three promising drug treatments (one drug to prevent cancer from spreading, another to treat blood cancer, and a third anti-cancer immunotherapy drug) developed in China which are close to regulatory approval for use in the USA. If approved, it is proof of China’s increasing ability to produce innovative treatment, and would be part of its wider economic transformation as it moves into higher-value and increasingly complex sectors. In the meantime, access to quality drugs remains a problem for the wider Chinese population, although some of China’s pharmaceutical companies are beginning to address this. (*New York Times*, 3 January 2018)

➢ In February, Bejing’s health authority notified all local hospitals that it would call off family/replacement blood donations, with near-immediate effective (family/replacement donations are blood donors who donate only when required, to family or community members). Donating blood in China is voluntary and unpaid, and a high proportion comes from family/replacement donations. China’s demand for blood exceeds supply – according to the World Health Organization, 0.92% of China’s population donates blood, but 1-3% is needed to maintain adequate blood supplies. Despite legislation banning the sale of blood, the family/replacement system has led to an illegal market in paid donations whereby agents sell blood to desperate families, and the decision by Beijing’s health authority was supposedly to tackle this. However, as a result, many patients were left without essential blood donations, leading to appeals for donations through social media, and for calls on the health authority to re-think its stance. Moreover, many doctors claim that they were not consulted before the change was introduced. (*The Diplomat*, 15 February 2018)

➢ Sanming, a city of 2.5 million people in China's Fujian province, is tackling health care cost inflation by taking on pharmaceutical companies and local doctors. From 2004 to 2014, health spending in China tripled – outstripping income growth – as an ageing population is more susceptible to non-communicable diseases, and hospitals have met shortfalls in funding by selling branded drugs at steep mark-ups. With limited state-funded health care insurance, patients must meet more than 33% of costs by themselves. Sanming is switching from premium drugs made by multinational pharmaceutical companies, to cheaper local generic drugs, and cracking down on doctors who take payments from distributors. Sanming combined hospital management, drug procurement and health insurance offices, linked the salaries of hospital managers to how much they cut costs; and stopped big mark-ups on drugs. The government insurance fund based reimbursement rates on the price of generics, and drug prices fell. Other regions are following Sanming's example, banning hospital mark-ups and securing aggressive sharp price cuts from multinational companies. However, reform has led to shortages of high quality drugs, and some doctors have left the profession. (*Financial Times*, 19 February 2018)

➢ Four years after the government’s resolution that “we will resolutely declare war against pollution as we declared war against poverty”, it appears than China is winning the war against pollution, with cities having cut the amount of fine particulates in their atmospheres by an average of 32% in that period. China fought air pollution by introducing a national air-quality action plan, which required all urban areas to reduce fine particulate pollution by at least 10%, with Beijing being required to have a 25% reduction. China prohibited the construction of new coal-fired power stations in its most-polluted areas; existing plants had to reduce their emissions, or replace goal with cleaner natural gas. Some cities restricted the number of cars on the road, cut iron and steel-making capacity, and closed coal-mines. These achievements in curbing pollution will ultimately improve the health and life expectancy of China’s citizens. If the declines in air pollution are maintained, it could add an additional 2.4 years to average life expectancy nationally, with more-marked gains in heavier-polluted cities. However, these improvements still leaves China’s air quality behind that recommended by the World Health Organization, and if China could meet these WHO targets, 4.1 years would be added to life expectancy. (*New York Times*, 12 March 2018)

➢ China has created a new super-ministry, replacing the China Food and Drug Administration (CFDA), and other health care agencies. The reform was approved by the 13th National People’s congress in March 2018. CFDA is the primary agency in charge of food, drug devices s and cosmetics regulation, and the chief health care regulator, the National Health and Family Planning Commission, have been merged into other agencies. Although the full details of the reform are still to emerge, it could have major implications for the future development of China’s food, drug, and health care regulatory regimes and policies, and is a significant re-organisation of China’s central government machinery. (*National Law Review*, 27 March 2018)

## Europe

➢ Statistics from the World Health Organization show that Russians consumed less alcohol per capita than French or German people in 2017, even when illegal alcohol is included. Tobacco smoking also fell sharply – a 25% decline between 2009 and 2016 – with 30% of Russians now smoking. Melita Vujnovic, the WHO's representative in Russia, credits this to legislation introduced over the past 13 years to combat Russia’s public health crises and increase life expectancy. In 1990, total alcohol consumption was under 12 L, but following the collapse of the Soviet Union it increased until 2007. Although tobacco prices are still below levels elsewhere in Europe, smoking in Russia is gradually becoming more socially unacceptable. In addition to government legislation, the rise in healthy living, particularly in cities, is another factor behind falling alcohol and tobacco consumption. (*The Standard*, 17 January 2018)

➢ A computer algorithm failure, dating back to 2009, may be responsible for the deaths of up to 270 women in the UK, under the UK’s National Health Service’s breast screening programme. Nearly 500 000 women aged between 68-71 years were not invited to their final breast screening appointment, due to computer software glitches. According to Mr Jeremy Hunt, the UK’s Health Secretary, estimates based on statistical modelling show that there may be between 135-270 women who have had their lives shortened as a result. There are a number of linked causes, including issues with IT and how women’s ages are programmed into the software. The problem came to light thanks to updates to the IT screening system to improve data insights, which revealed that some women were not receiving the invitation to their final screening at the age of 70. Mr Hunt promised all necessary steps to prevent a recurrence of this problem, and that the government is “united in our resolve to be transparent about what went wrong.” (*The Register*, 2 May 2018)

➢ Despite being home to the Mediterrean diet, widely perceived as being healthy, new data from the World Health Organization (WHO) shows that childhood obesity rates in the Mediterranean region of southern Europe are amongst the highest in the world. Of the 34 countries in Europe, Cyprus, Greece, Italy, Malta, San Marino and Spain had the highest rates, with 18-21% of boys being obese, and slightly lower rates amongst girls. This is higher than the USA, where 17% of children are obese, and twice as high in southern Europe than northern Europe. The WHO believes this increase in childhood obesity is due to the loss of traditional diet patterns, and the increased intake of sugars and energy-dense foods combined with low levels of physical activity. The Mediterranean diet is characterised by high intakes of fruit and vegetables, olive oil, moderate fish and poultry consumption, and low intake of dairy products, red meat and sweets, and is believed to support cardiovascular and metabolic health. Changing dietary patterns across southern Europe may be linked to increased poverty rates, and childhood obesity can increase the risk of obesity, diabetes and cardiovascular disease in adulthood. (CNN, 29 May 2018)

➢ Research on health care quality levels across Europe during the 2008 financial crisis has found varying levels of care during the course of the crisis. Whilst the financial crisis was felt across the world, not all countries experienced the same impact on health care. The researchers looked at mortality and morbidity rates for people living in 17 countries from 1980 to 2014, and 2002 to 2014 respectively. They also surveyed 350 000 people regarding their levels of health and quality of health care received. The research uncovered major differences across countries, with some western European countries undergoing a steady improvement in mortality rates for people in all income brackets – a trend which began before the crisis and continued thereafter. In eastern Europe, morbidity rates are largely unchanged for people with less income or education, although overall mortality rates did improve. In all countries that experienced the strongest financial shocks, people across all income brackets had reduced health care services. (*Medical Xpress*, 5 June 2018)

➢ The World Health Organization (WHO) has published an assessment on Europe’s mental health institutions and social care homes, covering 25 countries. It uncovered many problems, including that not one of the 75 institutions fully met all the UN standards on the rights of people with disabilities. It also found instances of patients sleeping on floors, insect infestations, and lack of basic facilities such as toilet doors, toilet paper and shower screens. Some institutions restricted patients’ communications with the outside world, and often did not respect their right to privacy. Often, there were no occupation or activities for patients, including learning and skills training. There were also failures in rehabilitating patients into the community. Each institution was assessed on 25 standards across five areas – the right to an adequate standard of living, the right to enjoy the highest achievable standard of physical and mental health, and freedom from torture, abuse and punishment. Although the study has limitations, its findings were described by Dr James Kirkbridge, a psychiatric epidemiologist at University College London said “… quite shocking that the level of care in some inpatient psychiatric settings in parts of Europe is so behind what we would expect a 21st century mental healthcare system to look like.” (*The Guardian*, 5 June 2018)

## India

➢ India has the largest number of young people – 600 million people aged under 25 – in the world. However, its sheer number of young people has yet to become an asset, with only 2.3% of India’s workforce having formal training in skills (compared to 96% in South Korea), and less than 25% of India’s graduates are immediately employable. Some estimates suggest that to create enough productive job, and enable India to dominate the global economy, it needs to build at least 1000 universities in the next 10 years, and nearly 50 times as many technical colleges. In the meantime, India’s young people combine traditional family values with American life goals – money and fame – and work in sectors such as online viral content and writing clickbait slogans for companies such as WittyFeed in Indore. Other people are drawn to working in call-centre scam, mainly because of the lack of other, secure, job options. (*The Guardian*, 13 January 2018)

➢ Despite India’s status as an emerging super-power, millions of its children are still malnourished, due to unhealthy diets, poor sanitation, and their mothers’ own bad health and low social status. According to India’s 2015-16 national family health survey, 38% of children aged under 5 years are stunted – although this a reduction from 48% in 2005-6, it is still much higher than other, poorer, countries. The survey also found that 21% of children aged under 5 years are wasted – a sign of recent acute hunger. The World Bank estimates that malnutrition costs India’s economy US$ 12 billion a year in lost productivity and higher health care spending. The government funds food programmes, but much of this assistance fails to reach its intended recipients, or the most vulnerable people (eg, in poorer families, young girls are often given less to eat than their brothers). Moreover, food programmes are cereal-based, which provides calories but not enough protein or micronutrients – and fresh fruit, vegetables, milk, eggs and meat are expensive. However, India’s government recently approved a national nutrition mission – a US$ 1.4 billion, 3-year initiative to tackle malnutrition through better integration and stronger monitoring of various government programmes. It has set aggressive targets for the reduction of stunting, anaemia, and low-birthweight babies. Purnima Menon of the International Policy Research Institute is optimistic. “The minute you really get people paying attention to what is happening on the ground and having reviews, things actually start moving,” she said. (*Financial Times*, 22 January 2018)

➢ The World Health Organization (WHO) has certified India’s first vaccine against rotavirus, allowing it to be purchases by agencies such as UNICEF and GAVI to tackle diarrhoea in other countries. The vaccine, manufactured by Hyderabad-based Bharat Biotech, is part of the universal immunisation programme across nine states, and will be rolled out in Jharkhand over the next 1-2 months. In India, the vaccine costs US$ 1/dose, and the low cost, combined with WHO certification, means that it can be considered by other countries as a cheaper option to tackle the burden of diarrhoea. Mr Krishna Ella of Bharat Biotech, confirmed that a UNICEF team is now discussing its purchase. Currently there are three rotavirus vaccines, from Merck, GSK and Bharat Biotech. A fourth vaccine that can protect against multiple strains of rotavirus is under development at the Serum Institute of India, Pune. According to a former WHO official, there is evidence the Bharat Biotech vaccines offers cross-protection against various strains of rotavirus. Whilst there are other Indian vaccines with WHO pre-qualification, this is the first rotavirus vaccine with WHO certification. (*Deccan Herald*, 24 January 2018)

➢ India’s Union Budget of 1 February 2018 announced the launch of the National Health Protection Scheme (dubbed “Modicare”), which aims to extend health care insurance to 100 million families, and to increase the insurance ceiling to Rs 5 lakh (US$ 7760) per family. Early estimates suggest that it will cost US$ 1000 billion. The scheme will target up to 500 million people from low-income households (41.3% of India’s population), who will be identified from the 2011 socio-economic caste census. A national health agency will be set up to oversee its implementation at the state-level, and the extended coverage will also require more medical colleges and health professionals – India already has a shortfall of doctors per head of population. “India cannot realise its demographic dividend without its citizens being healthy,” said the Finance Minister, Mr Arun Jaitley. (The Hindu, 2 February 2018)

➢ Following an outbreak of the nipah virus in Kerala, emergency measures have been imposed, including bringing in health experts to help contain it. Nipah is a rare virus, but it has a mortality rate of 70%, and the World Health Organization lists it alongside Ebola and Zika as 1-of-8 priority diseases that could cause a global epidemic. Pigs were the host in previous outbreaks of nipah, but fruit bats appear to have caused the spread of this current outbreak. According to local media, nearly 200 people are receiving hospital treatment, 26 people are under observation and three are under intensive care. The union health ministry believes that this outbreak should remain localised, but are advising people to take precautions, including avoiding fruit that has fallen to the ground or is blemished, and also advises against travelling to affected states. (Sky News, 28 May 2018)

## The Americas

➢ Research published in *Health Affairs* shows that children in the USA have a 70% higher chance of dying before adulthood compared to children in other developed countries. The research reveals the USA’s gaps in child health outcomes, which has led to an estimated 600 000 excess child deaths since 1961. The study was published after Congress’s decision to end the Children’s Health Insurance Program funding – the programme provided insurance to 9 million low-income US children. It also builds on data released earlier in 2018, which shows that overall life expectancy in the USA has fallen over the past two years. The research compared the US to 19 other countries (including France, Canada, Australia and the UK), and found that the risk of death in the US was 76% higher for infants, and that its infant death rate from extreme prematurity was three times higher than comparator countries. Dr Askhish Thakrar, one of the researchers, argues that the some of the health outcome gaps are related to the US’s fragmented health care system and Medicaid health insurance often only applying after pregnancy is confirmed may lead to untreated health issues. The rise in child poverty in the 1980s also coincides with the US falling behind comparator countries on health outcomes. (Vox, 8 January 2018)

➢ Porto de Careiro da Várzea, a small town close to Manaus, Brazil’s jungle capital, is mostly known as the starting point for route BR319, which connects the city to the rest of the country. However, the town is also at the centre of Brazil’s rising obesity problem, and Brazil’s Amazonian cities, once plagued by hunger, are the overweight capitals of one of the developing world’s most obese countries – an estimated 21% of Brazil’s population aged 15 or over was obese in 2015. Brazil’s obesity levels rose 60% from 2006 to 2016, with 9% of children aged 5-10 years considered obese in 2008 – by 2016, this had risen to 13%. Across Brazil, although obesity rates have remained largely static for children aged under 5 years, they have increased from 6.5% to 8% in the Amazonian states. Nutritionists believe that increasing obesity amongst Amazonia’s children is partly due to improved social welfare benefits, which have reduced poverty, but some families have used some of the cash to buy soft drinks and fatty foods instead of natural foods, which are also more expensive than processed food. (*Financial Times*, 22 January 2018)

➢ According to the UNAIDS Director for Latin America and the Caribbean, Dr Cesar Núńez, 1-in-3 people living with HIV in the Caribbean do not know their status. Speaking at the 6^th^ Meeting of the National AIDS Programme Managers and Key Partners, he also reported that at the end of 2016, 81% of people who knew their status were accessing treatment. However, he highlighted the problems of late diagnosis of several countries in the Caribbean – particularly amongst men – and that 33% of people receiving treatment for HIV did not have viral suppression; and that the region as a whole is lagging behind in terms of testing and viral suppression targets. (Newsday, 12 March 2018)

➢ Over the past few months, about 80 politicians in Mexico have been murdered, and the country’s murder rate in 2017 was the highest in decades, with up to 97% of the country’s murders being unsolved. Officials blame organised crime for politicians’ deaths, with reasons such as voter intimidation, revenge on leaders who align with rival criminal organisations or stand up to cartels. National law-makers are protected by bodyguards and work in the relatively secure Saint Lazarus Legislative Palace, but local leaders lack such protection, and 1972 local politicians have been killed since 2006. The Mexican newspaper *Milenio* has reported that the national electoral institute has received calls for help from candidates running for parliament, but it will not be possible to protect every candidate. Investigative journalist Jose Reveles estimates that 45% of all local government in Mexico is controlled by organised crime. “Narcopolitics isn’t some vague threat,” he said. “It’s a reality.” (*Deutsche Wells*, 22 April 2018)

➢ Thanks to genome sequencing, people in Canada must cope with the implications of being diagnosed with an inheritable disease alongside the more practical concern of the impact of test results on insurance premiums. However, the Genetic Non-Discrimination Act (GNA) came into force in May 2017, making it illegal to require individuals to disclose genetic test results or undergo genetic tests for any agreement or service. However, this has been challenged by the Quebec provincial government. This has thrown the GNA into danger and prompted a defence by scientists who argue that it empowers people to take genetic tests, and therefore take preventative measures to reduce disease risks. Whilst the legislation on testing has stalled, diagnostic technology using gene sequence marches on, including tests for pre-natal Down Syndrome and multi-gene cancer testing. (*Technology Networks*, 23 May 2018)

